# Grandmaternal microchimerism: interesting curiosity or clinically relevant phenomenon?

**DOI:** 10.1016/j.ebiom.2021.103743

**Published:** 2021-12-09

**Authors:** German Tapia

**Affiliations:** Department of Chronic Diseases, Norwegian Institute of Public Health, Oslo, Norway

Maternal microchimerism, the presence of a small number of maternal cells in the offspring, usually results from transfer of maternal cells during pregnancy, [1] or possibly during breastfeeding [2]. In 1999, the group of J. Lee Nelson demonstrated that microchimerism of maternal origin persists into adult life [3]. Maternal microchimerism seems to be evolutionary conserved, which suggests that maternal microchimerism has an evolutionary advantage and could be important for reproductive fitness and possibly disease [4].

In the current issue of *EBiomedicine*, Karlmark et al. present novel data indicating the presence of grandmaternal microchimerism in fetal cord blood [5]. This is the first time grandmaternal microchimerism has been presented as more than a theoretical possibility. The detection of grandmaternal genetic material in minute amounts calls for a meticulous methodological approach, the use of very specific and sensitive laboratory methods, and a study population consisting of multigenerational families. Working from an existing cohort of primigravid women and their offspring, Karlmark et al. detected DNA compatible with grandmaternal microchimerism in five of 28 cord blood samples, using HLA sequence-specific real time PCR. Karlmark et al. found grandmaternal microchimerism to be present in much lower quantities (∼100 fold lower) and be less prevalent than maternal microchimerism in the same cohort (18% vs 57%, respectively) [5].

In this pioneering study, the number of study participants was limited, and only a few samples were positive for grandmaternal microchimerism. This is not surprising given the low quantities detected, and that three generations with specific HLA-genotype combinations are needed, which quickly winnows down the eligible participants from the study population. The low number of positive samples is also not surprising, as very low quantities of grandmaternal genetic material were reported. Theoretically, there could be other sources of the detected DNA. Undetected prior miscarriages, unreported blood transfusions, laboratory errors or other, unknown sources of DNA could all lead to detection of non-self, non-maternal DNA in small amounts. Yet, it is hard to envision other possible sources of microchimerism being behind these findings, as these are rare events and it seems improbable all positive samples would be from other sources and have correct HLA genotype by chance. Still, the findings of Karlmark et al. should be replicated to firmly establish the presence and prevalence of grandmaternal microchimerism is in larger number of subjects, and ideally also using other methods to detect grandmaternal cells.

Microchimerism is a field of study that has profound implications for immunity and evolution. Although there are still significant gaps in our knowledge, maternal microchimerism has added another layer to the already complex tapestry of genetics, epigenetics and environment. Grandmaternal microchimerism could further deepen the complexity in this interplay. Although the clinical and biological significance of grandmaternal cells in cord blood for the individual is unknown, and the levels and prevalence are much lower than that for maternal microchimerism, it is not inconceivable that they too could have a similar clinical relevance. Among the potential clinical implications of maternal microchimerism are postnatal tolerance to non-inherited maternal antigens (NIMA), with improved long-term graft survival and less severe graft-vs-host disease observed in NIMA-matched donors [5,6]. Maternal microchimerism has also been suggested to play a role in pregnancy [4,7]. Future studies should investigate whether grandmaternal microchimerism could have similar associations to pregnancy outcomes, and if the offspring has any tolerance against non-inherited grandmaternal antigens. Another topic of future studies is whether grandmaternal cells are present in breastmilk. The immunological properties of maternal microchimerism could be potentiated or linked to breastfeeding, [2] which might suggest that grandmaternal microchimerism, even if present in cord blood, would not have similar immunological properties unless it is also present in breastmilk. Maternal microchimerism has also been hypothesized to be involved in offspring response to pathogens, [8,9] and autoimmunity (reviewed in eg, [1,6]). Yet, maternal microchimerism in cord blood at birth is not necessarily associated with later autoimmunity, [10] and whether maternal microchimerism is involved in pathogenesis or healing in still unknown [1]. Karlmark et al. detected grandmaternal HLA sequences in whole blood, peripheral blood mononuclear cells and CD34+ cells, but these are not necessarily viable cells in the fetal tissues or circulation, and whether these grandmaternal cells persist in childhood, or to adulthood, or are quickly cleared from the fetus is an open question that should be answered. A longitudinal study with both cord blood and serial postnatal samples would help to clarify this.

Several decades after the first human studies of maternal microchimerism, technological advances continue to advance the field, but there are still many open questions. The study of grandmaternal microchimerism has now begun, and only time will tell if there are any associations to immunity, fitness, disease and health.

## Contributors

GT conceived and wrote this invited Commentary.

## Declaration of Competing Interest

The author has no conflicts of interest to disclose.
